# An Integrated Approach to Optimizing Cellulose Mercerization

**DOI:** 10.3390/polym12071559

**Published:** 2020-07-14

**Authors:** Monica Ferro, Alberto Mannu, Walter Panzeri, Con H.J. Theeuwen, Andrea Mele

**Affiliations:** 1 Department of Chemistry, Materials and Chemical Engineering “G. Natta”, Politecnico di Milano, Piazza L. da Vinci 32, 20133 Milano, Italy; albertomannu@gmail.com; 2 CNR–SCITEC, Istituto di Scienze e Tecnologie Chimiche, Via Alfonso Corti 12, 20133 Milano, Italy; walter.panzeri@polimi.it; 3 Nouryon Chemicals bv, Westervoortsedijk 73, 6827 AV Arnhem, The Netherlands; con.theeuwen@nouryon.com

**Keywords:** Cellulose, mercerization, XRD, PCA, DoE

## Abstract

An integrated approach, based on quantitative transmission mode powder X-ray diffraction (PXRD) combined with multivariate statistical analysis, has been applied to cellulose obtained from three different sources to correlate the mercerization degree and crystallinity with the cellulose type, temperature, and reaction time. The effects of the experimental conditions on the two outcomes were studied by design of experiments (DoE) and surface responding analysis (SRA) combined with principal component analysis (PCA). SRA showed a marked influence of the type of cellulose (wood cellulose from the kraft vs. sulfite process, WCK vs. WCS) on the conversion of cellulose I to cellulose II (CII%) during mercerization. A counterintuitive simultaneous effect of temperature and cellulose type was also highlighted. The data elaboration in the form of response surface plots provided an easy predictive tool for the optimum conditions to maximize the conversion. The simulation reported for WCK showed maximum conversion (96%) at 70 °C in 24 h with 18%wt NaOH.

## 1. Introduction

Cellulose, the most abundant polysaccharide in nature, is formed of anhydroglucopyranose (AGU) repeat units linked by β(1→4) glyosidic bonds. Cellulose can be obtained from several plant fibres and through the delignification of woody plants [1]. It is characterized by a complex supramolecular structure that is known to affect both the reactivity and macroscopic properties of the cellulose polymer [2]. Native cellulose is a mixture of two crystalline forms, Iα and Iβ. Cellulose Iα has a triclinic unit cell containing one chain (P1 space group) and is present in algae and bacteria, while cellulose Iβ has a two-chain monoclinic cell (P21 space group) and is found in higher plants [3].

Cellulose is considered the main renewable source of C atoms as an alternative to fossil fuels. Cellulose is also the starting material for several classes of derivatives, which are mainly produced from dissolving-grade wood pulps (hardwood and softwood) containing hemicellulose and small amounts of lignin. To a lesser extent, cotton linters are used when a refined pure raw material is required with high cellulose content, low hemicellulose and lignin contents, and homogeneous molecular weight distribution [4,5,6]. According to the literature [7], cotton and wood cellulose contain predominantly the Iβ form, which, for clarity, will be referred to hereafter as cellulose I (CI). In addition to CI, crystalline modification cellulose II (CII) is important industrially because it is the starting material for the preparation of many cellulose derivatives, such as viscose [8] and cellulose ethers and esters [9]. The most important contributions to the global production of cellulose derivatives come from cellulose acetate, used in coatings and membranes [10], cellulose xanthate, used in textiles [11] and carboxymethylcellulose [12], used in coatings, paint, and pharmaceuticals [13,14].

**Figure 1:**
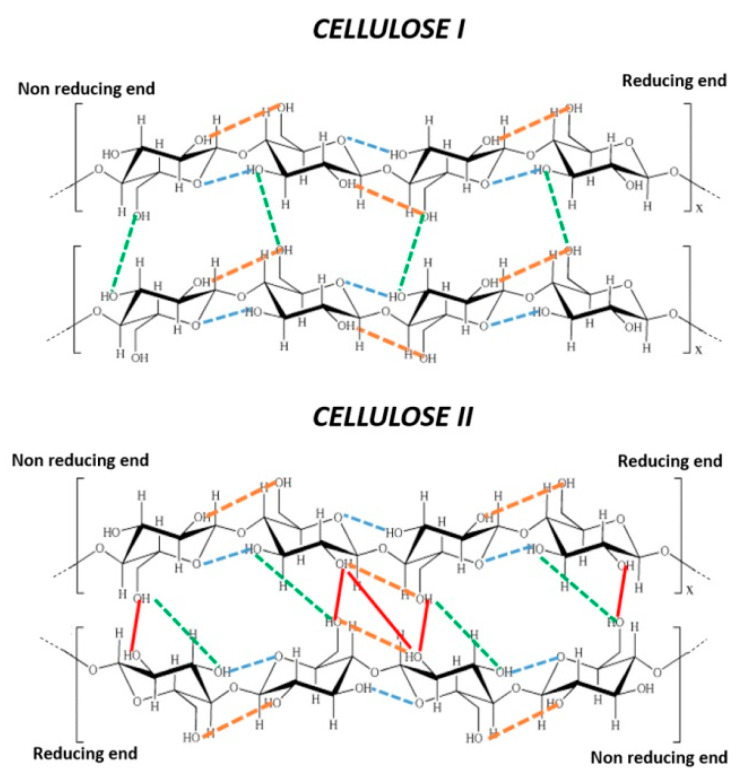
Intermolecular and intramolecular hydrogen bonds in structures CI and CII: Intramolecular 2(OH) … O-6 (yellow dashes), intramolecular O(3)H–O pyranosidic (blue dots), intermolecular O(6)H–O(3′) (green dashes and dots), and intermolecular O(2)H–O(2) and O(6)H–O(2′) (red lines).

Cellulose chains tend to aggregate by forming an ordinate network of intermolecular hydrogen bonds, as Figure 1 (green and red lines). Additionally, each cellulose chain of both CI and CII has limited conformational flexibility for the presence of two types of intramolecular hydrogen bonds: (i) those connecting O2–H hydroxyl groups to the O atom of the primary OH groups of the neighbouring AGU (Figure 1, yellow lines) and ii) those connecting O3–H to the pyranosidic O atom (O6) of neighbouring AGU (Figure 1, blue lines) [15].

**Figure 2:**
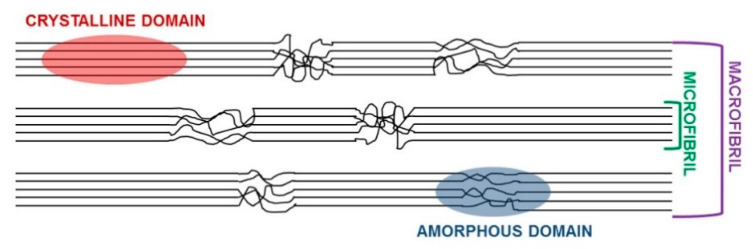
Cellulose fibrils in CI. Highly-ordered regions (crystalline domain) and disordered regions (amorphous domain) are shown.

These structural motives provide CI with a compact structure that makes cellulose, in its native form, recalcitrant to reactions. To convert native cellulose into cellulose derivatives, good cellulose accessibility and reactivity are desired [16]. The accessibility of CI, namely, the possibility for reactants to reach and react with free OH groups on the polysaccharide backbone, leading to cellulose derivatives, depends on: (i) surface area, as determined by the size of the accessible cellulose fibril aggregates, (ii) cellulose macromolecular structure, which determines the hydroxyl groups that are accessible and (iii) size and type of reagent used during derivatisation. The accessibility of the fibril surface or fibril aggregates is limited by the compact structure of CI, which is determined by the presence of highly ordered regions formed by strong hydrogen bond networks [17] (Figure 2). The reactivity of cellulose can refer to its capacity to undergo diverse chemical reactions. Accordingly, accessibility is a necessary, but not sufficient, condition for efficient cellulose derivatization. Each AGU in a cellulose chain has three different types of hydroxyl groups (Figure 1), with hydroxyl groups O(2)H and O(6)H as the main reactive groups that are susceptible to chemical attack and functionalization [18].

Several studies have attempted to overcome the problem of cellulose recalcitrance to chemical modifications. However, due to the complexity of the cellulose structure, many aspects remain to be considered altogether, such as wood species, morphology and pulping process [19]. The unambiguous and quantitative evaluation of cellulose accessibility is difficult because it depends on several factors, such as particle size, degree of polymerization (DP), crystallinity and cellulose purity (presence of hemicellulose or lignin). Alkali treatment is a well-known approach to cellulose activation, and is commonly referred to as mercerization [20,21,22,23,24]: in this process, the more reactive and thermodynamically stable CII is formed from CI. When cellulose interacts with NaOH, Na^+^ cations penetrate intracrystalline spaces, causing the cellulose to swell. Consequently, intermolecular hydrogen bonds (Figure 1) are broken. After washing, neutralizing and drying, cellulose undergoes an irreversible morphological and structural change to CII that is characterized by an antiparallel chain motif. This latter structural detail was confirmed by Langan et al. at 1 Å resolution [25]. This structure is stabilized by a network of intermolecular hydrogen bonds of type O2-H---O6, O6-H---O6, and O2-H---O2 (Figure 1) [26,27]. Some hydroxyl groups that are inaccessible in the CI crystalline form become accessible in the more amorphous CII form. The number of available hydroxyl groups in CII is increased by around 25% compared with CI, [28,29] making mercerization a fundamental step for cellulose activation toward further transformations. As previously reported, the polymorphic transformation of CI into CII is initiated at a sodium hydroxide concentration greater than 7–8 wt% [30,31,32,33], but mercerization probably already starts at low NaOH concentration (between 5–7 wt%) and low temperatures (near 0 °C) depending on the cellulose source [34,35].

The mercerization process has a non-negligible environmental impact [36,37], as treating cellulose with strong alkali produces a large volume of dilute sodium hydroxide solution waste. This is toxic to wildlife and cannot be discharged into groundwater for economic and ecological reasons. An important approach to sustainability involves reducing the energy and chemicals used in this process. In principle, this could be achieved by optimizing the mercerization process parameters. This study aims to develop a general model based on analytical and statistical data to select the optimum mercerization parameters. The present work does not aim to estimate the best mercerization conditions that are already widely studied in the literature, nor the thorough characterization of the interplay of CI and CII in the merceritazione process, recently investigated via both PXRD and solid state ^13^C NMR spectroscopy [38,39]. Rather, the purpose of the work is to provide analytical and predictive tools that allow to optimize the mercerization reaction in the range of time and temperature mimicking that of the actual industrial production carried out in real plants. To this end, we report on lab scale mercerization tests done on different types of cellulose and exploring the time and temperature range compatible with that used in typical cellulose derivative production [40].

Three commercially- and industrially-relevant cellulose types were considered, namely, cotton linters cellulose (CLC), wood cellulose obtained from the kraft process (WCK) and wood cellulose obtained from the sulfite process (WCS). Mercerization is strongly dependent on the reaction conditions, such as temperature, NaOH concentration, and reaction time [41,42,43]. Cellulose samples were treated on a laboratory scale by using 18 wt% NaOH solution at different mercerization times and temperatures. Morphological and structural changes were studied by scanning electron microscopy (SEM) and transmission-mode powder X-ray diffraction (PXRD), respectively. For the latter measurements, tailored and novel sample preparation is presented herein. The proposed protocol has two main advantages: (i) Pellet sample preparation significantly improves the quality of transmission mode data by reducing the effect of air scattering, which is known to lead to spectral noise or problems with the baseline due to a diffuse background; [44] and (ii) the spectrum obtained in transmission mode shows more defined peaks in the region of 2θ = 25–40° due to the preferred orientation, generally referred to as texture, of well-oriented cellulose fibrils. In particular, we showed that the reflection due to the 004 crystalline plane at 2θ = 34.8° can be conveniently exploited (vide ultra) as an experimental descriptor of CI conversion to CII. Notably, this diffraction peak was hardly detected in reflection mode.

Cellulose reactivity was also evaluated by multivariate analysis (principal component analysis, PCA) of the PXRD data. The effects of temperature and time on different celluloses were evaluated using a statistical approach by DoE analysis. The results of the present work provide information on the influence of reaction parameters on the quality of the treated cellulose. This represents the starting point for both process optimization strategies and the formulation of new strategies. Furthermore, the method presented was conceptualized and set up to avoid incomplete or excessive mercerization, which result in unwanted insoluble fractions and wasted time, energy and reagents (NaOH and cellulose). From this standpoint, and considering the scenario of a real-time quality control in the production line, it is important to stress that PXR diffraction represents an ideal source of input data for both multivariate analysis and DoE, for the accuracy of the data, the running costs of a standard powder diffractometer for routine analysis and the measurement time per sample.

## 2. Materials and Methods

### 2.1. Materials

Cellulose samples (WCS, CLC, and WCK) were supplied by Akzo Nobel Chemicals S.p.A. Novara (Novara, Italy) and milled to a maximum particle size of 500 μm.

The reported composition of celluloses was provided by Innovhub (Milano, Italy) according to the National Renewable Energy Laboratory protocol (NREL/TP-510-42618 2008) [45]. The relative error on mass determination was in the range 2–4%.

The mass values were then converted in%wt leading to the following compositions:

WCS from softwood: 88.7% glucose, 8.6% mannose.

WCK from softwood: 80.9% glucose, 5.8% xylose, 4.1% arabinose, 9.2% mannose.

CLC from cotton linters: glucose ≥99%.

### 2.2. Mercerization Protocol

Cellulose mercerization was performed in a 50-mL Erlenmeyer flask. Powder milled cellulose (200 mg) was completely soaked with NaOH solution (4 mL, 18 wt%) and allowed to react at different temperatures (rt, 40 °C, 60 °C, and 80 °C) for different contact times (15 min, 30 min, 1 h and 48 h). After 48 h, mercerization was considered complete, with the sample taken as the reference for totally mercerized cellulose. After treatment, the mercerized samples were washed to neutral pH with deionized water. The sample was then filtered using a Buchner funnel and the residue was air-dried overnight. An example of morphological analysis of cotton linters cellulose is discussed in Section 3.3.

### 2.3. Wide-Angle X-Ray Diffraction: Sample Preparation and Data Collection

A novel, simple and efficient method for the characterization of cellulose and evaluation of CI conversion to CII is proposed herein. Our approach was based on innovative sample preparation for PXRD and the use of X-ray diffraction data collected in both reflection and transmission mode. PXRD samples were prepared as compact pellets of compressed cellulose powder. The pellets, mounted onto tailored sample holders, were then examined in both transmission and reflection geometry. Reflection mode is commonly used for PXRD data collection, [46,47,48], while data collection in transmission mode is less common [49,50], although it has already been used for cellulose characterization [51]. Cellulose pellets were prepared by pressing milled cellulose (150 mg) with a KBr die set usually used to prepare KBr pellet samples for FT-IR analysis. The pellet was used for transmission mode PXRD data collection instead of the usual powder sample by placing in the sample holder of the diffractometer. This procedure improved XRD sensitivity by providing PXRD spectra with better signal-to-noise (S/N) ratios. The pressure applied did not alter the crystal structure of the cellulose sample.

Cellulose Iβ and II reflections were indexed according to French [52]. Data analysis was performed with Fityk 0.9.8 [53]. Fityk is an open-source software developed by Marcin Wojdyr for nonlinear fitting of analytical functions (especially peak-shaped) to data (usually experimental data). It is used in crystallography, chromatography, photoluminescence and photoelectron spectroscopy and infrared and Raman spectroscopy. Peak deconvolution was carried out with the following method: the baseline points were added manually, Gaussian peaks were added in the positions corresponding to the most prominent reflections and to the amorphous region in order to optimize the total fitting curve. The main crystalline reflections (−110, 110, 102, 200, 004) were fitted with five different Gaussian curves.

The crystallinity index (C.I.%) was evaluated from PXRD diffractograms obtained in reflection geometry using a peak-fitting procedure [54,55,56] as performed by Fityk 0.9.8 software. The C.I.% was calculated as the ratio of the area of crystalline peaks to the total area. Some examples of deconvolution are reported in Figures S1 and S2.

PXRD data collection was performed on a Bruker D2 Phaser X-ray powder diffractometer using CuKα radiation. Data were collected in the 2θ range of 4.7–40° using the following parameters: Step size, 0.02°; counting time, 0.4 s per step; primary slit module, 0.6 mm; air scatter screen module, 1 mm; and secondary slit module, 8 mm.

### 2.4. Principal Component Analysis

PXRD diffractograms obtained in transmission mode were subjected to principal component analysis (PCA). The two main advantages of this technique were that no calculations needed to be performed on the spectra and the full PXRD diffractogram could be used as the input for PCA analysis without any manipulation (known as binning). PCA was performed using the entire spectral region of 2θ = 4–40°. Normalization and Pareto scaling were applied. Spectral data in the ASCII format were imported into online tool Metaboanalyst 3.0. [57].

### 2.5. Design of Experiments

Data acquired through PXRD collection were subjected to multivariate analysis employing a two-level full factorial n^k^ model (*n* = 2 and K = 3) [58]. Three independent variables k, namely, the specific process (type of cellulose used), temperature and time, were optimized to study the response “conversion”, which corresponded to the degree of mercerization. Statgraphics Centurion v15.1.02 software (Statpoint Technologies Inc., The Plains, VA, USA) was used for experimental design data analysis and to develop the response surface. All statistical analyses were performed by comparing data with the unpaired Student’s t-test. The normal distribution of the data was checked by the Kolmogorov–Smirnov and Shapiro tests. *p* < 0.5 was considered to be statistically significant.

### 2.6. Scanning Electron Microscopy

The morphology of cellulose before and after mercerization treatment was examined by Cambridge stereoscan SEM S-360. The following instrumental parameters were used: high voltage: 10 kV, tilt: 0.00. The powdered samples were coated with a thin layer of palladium/gold and carbon cement was used as adhesive.

## 3. Results and Discussion

### 3.1. Structural Characterization

**Figure 3:**
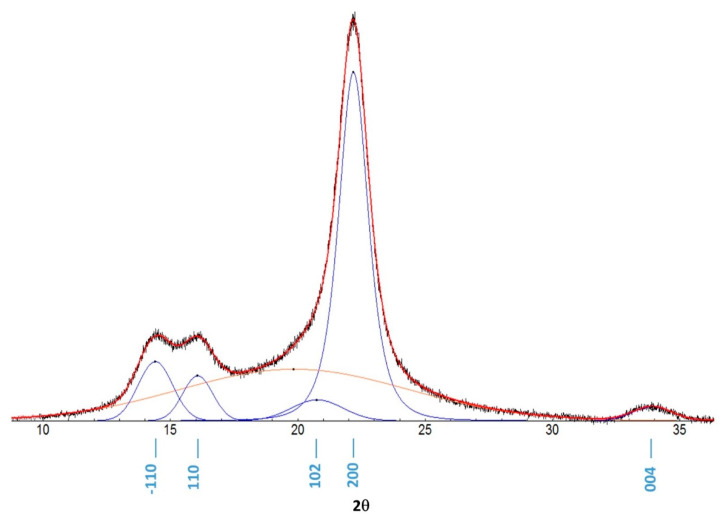
XRD peak deconvolution of CLC: the black line is the experimental diffractogram, the red line is the total fitting, the blue Gaussian peaks represent the main reflections and the orange Gaussian curve represents the amorphous region.

Structural characterization was performed by wide-angle X-ray diffraction of the powder samples. After data collection, the peak deconvolution routine was applied to the entire spectrum. An example of deconvolution is shown in Figure 3. The inclusion of further gaussian functions corresponding to minor reflections (e.g., 211, 013, −113, −112) resulted in unrealistic fitting characterized by overemphasis of the amorphous halo and physically meaningless negative Gaussian contributions in the 22–35° 2θ region. A graphic representation is present in the Supplementary Information as Example 1 and Example 2. Peak parameters were then exported.

**Figure 4:**
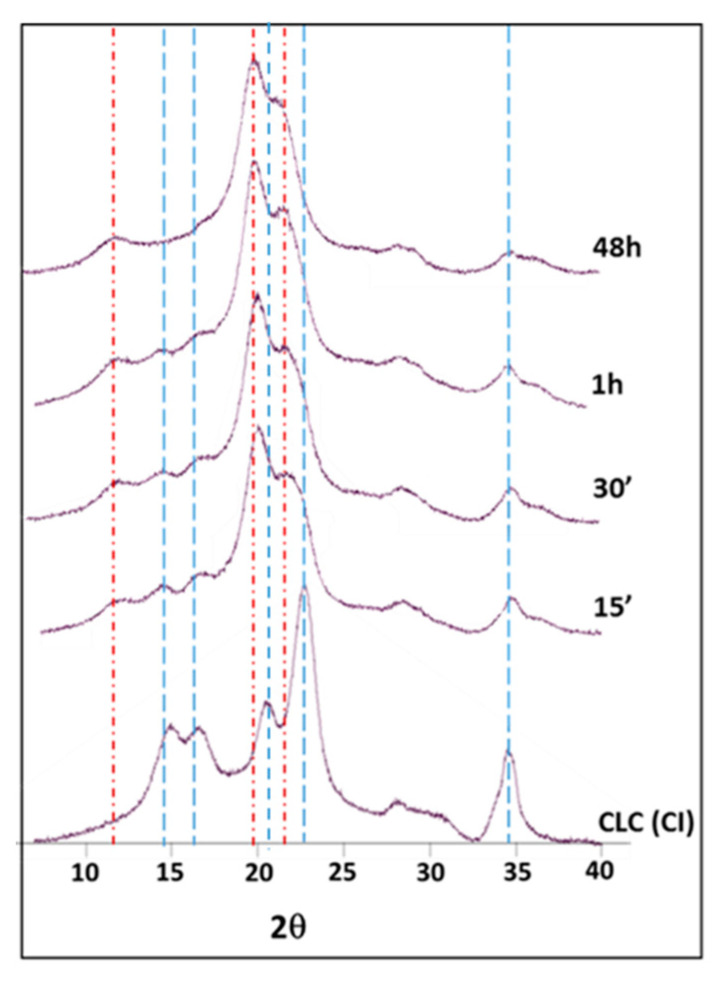
PXRD profiles of CLC at different mercerization times with constant NaOH concentration. Top trace is the reference for complete mercerization (see text). For clarity, the main peaks of CI [(1–10), (110), (102), (020) and (004)] and CII [(1–10), (110) and (020)] are indicated with blue and red lines, respectively.

The conversion of CI to CII (CII%) was evaluated from the experimental PXRD profiles [25,52,59,60]. Cellulose samples were treated with 18 wt% NaOH for 15 min, 30 min, 1 h and 48 h. The stacked PXRD profiles of cellulose obtained in transmission mode are shown in Figure 4.

The diffractograms showed changes in intensities of different peaks and a polymorphic transformation with increasing mercerization time. In particular, a decrease in the intensity of characteristic peaks of CI (14.7, 16.8, 22.7 and 34.8°) was observed after 15 and 30 min. Meanwhile, an increase in the intensity of peaks belonging to CII (12.1, 20.1 and 21.9°) was clearly detected [61] (Figure 4).The XRD diffractograms of CI and CII are reported in the Figure S3.

After 1 h, the typical peaks of CI had disappeared almost completely, and the resulting spectrum was similar to that of the sample mercerized for 48 h. This suggested that the mercerization process of cellulose was almost complete after 1 h, with the optimal reactivity of cellulose almost reached. When using transmission-mode data collection, the peak corresponding to the 004 plane at 34.8° was clearly visible and isolated, and could be used as a descriptor of the conversion of CI to CII [62]. During mercerization, the intensity of this peak decreased, indicating a different chains packing. The conversion from CI to CII was evaluated by quantifying the decrease in the area of this peak (A_004_) with respect to the starting cellulose (A_004cel_) according to Equation (1):

(1) $$\mathrm{CII}\%\;=\frac{A_{004}}{A_{004\mathrm{cel}}}\cdot 100$$

**Figure 5:**
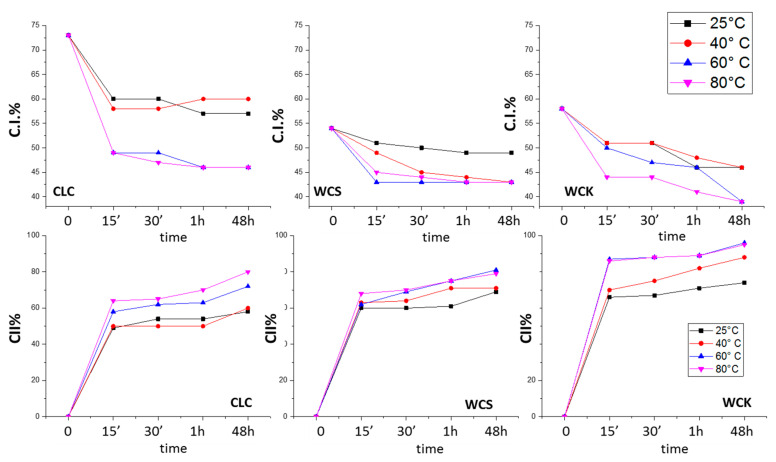
Trends in the crystallinity index (C.I.%, top row) and conversion (CII%, bottom row) of cellulose I to cellulose II at different mercerization times for CLC, WCS and WCK at different temperatures.

The degree of crystallinity and CII% values of the three celluloses as a function of the mercerization time and at different temperatures are reported in Figure 5. The C.I.% of the examined cellulose samples show a trend in line with what reported by Revol [63], i.e., the C.I% depression is dependent on the cellulose source.

The data of Figure 5, in particular, indicate a marked loss of crystallinity in the case of CLC, which takes place in the first 15 min of contact with NaOH. This effect was amplified at high temperatures (60 and 80 °C), where the crystallinity reached values close to those typical of wood cellulose WCS and WCK. Conversely, at 25 and 40 °C, CLC maintained a high degree of crystallinity (>55%). Similar behaviour was observed for WCS and WCK. Again, a major loss in crystallinity was observed in the first 15 min, which was more evident with increasing temperature. However, the maximum crystallinity loss detected for WCS and WCK was 15%, compared with 25% for CLC.

The three types of cellulose showed different trends in the CII% value (Figure 5). This indicated a difference in reactivity, which was attributed to the characteristics of cellulose studied, summarized in Table 1. The data reported in Table 1 indicate that: (i) Cellulose from cotton linters (CLC) is characterized by high purity, high DP, and high C.I.%; (ii) WCS obtained from wood via a sulfite process results in a higher crystallinity with respect to other wood celluloses, higher purity, and a relatively low DP; and (iii) WCK obtained from wood cellulose by the kraft process has a low DP, low C.I.% and contains approx. 16% hemicellulose.

The comparison of the curves in Figure 5 (bottom row) showed that WCS conversion during mercerization was hardly affected by changes in temperature, while the CLC conversion gradually increased with increasing temperature. WCK conversion was sensitive to temperature in the range of 25–60 °C, with no further improvement in conversion observed above 60 °C. All C.I.% and CII% values are reported in the SI (Tables S1–S3).

As mentioned previously, the reactivity of cellulose resulted from the correlation of several parameters. This makes studying cellulose reactivity using a one-factor-at-a-time approach difficult. Furthermore, effects related to the concomitant variation of more than one parameter on the system cannot be analysed using classic 2D plots. Accordingly, multivariate statistical approaches, namely, PCA and DoE, were used to evaluate the effect of multiple variations of different parameters on the type of process, conversion, and loss of crystallinity.

### 3.2. Morphological Characterization

**Figure 6:**
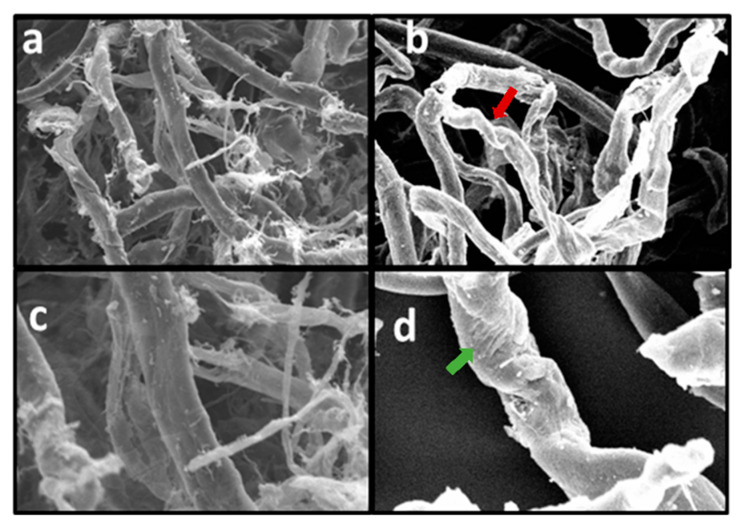
SEM images of CLC sample before mercerization treatment at 400× magnification (**a**), 1000X magnification (**c**) and after mercerization treatment at 400× magnification (**b**) and 1000× magnification (**d**).

The SEM images reported in Figure 6 report the evolution of the morphology of the CLC fibres before and after mercerization [64,65]. The morphological changes after NaOH treatment can be summarized as follows: before the treatment, the fibres resulted thin and straight, and showed the typical ribbon-like of native cellulose (Figure 6a).The fibres have an average diameters of 8–15 μm [62] and a finer network of microfibres 1–2 µm in diameter was found among fibres. These finer fibres are no longer visible after mercerization (Figure 6b) probably because of a re-aggregation of the larger fibres during the alkaline treatment [62]. After mercerization, cellulose fibres were slightly thicker and twisted (Figure 6b, red arrow). The diameter of the fibre are larger than the fibres before the treatment (15–20 μm). The changes observed by SEM confirm the swelling of the fibres in alkali and the shrinkage due to the rearrangement of the fibres. At a larger magnification (1000×) differences in the fibres’ surfaces can be also detected. The native cellulose presents a smooth surface (Figure 6c), while mercerization shows a rough surface (Figure 6d, green arrow) [48,66]. These differences in morphological structure and fibre surface were observed also in WCK and WCS samples.

### 3.3. Principal Component Analysis

**Figure 7:**
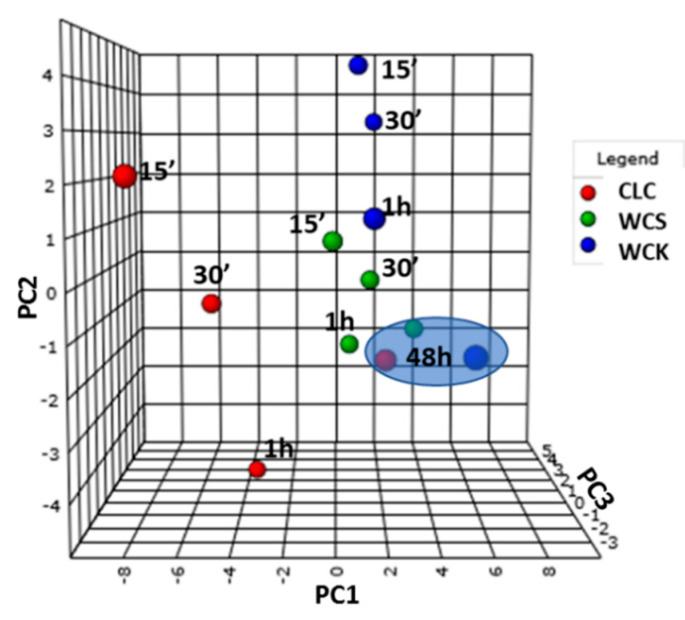
3D PCA scores plot of mercerization at 80 °C.

To achieve a fast and unbiased classification of samples in terms of reactivity, PCA was applied to the PXRD diffractograms in transmission mode [67,68]. The score plot of mercerized cellulose samples at different mercerization times (15 min, 30 min, 1 h, and 48 h) is shown in Figure 7. Each data point in the score plot represents a spectrum. As the proximity of points indicates similarity, scores closer to the mercerized samples (blue ellipse) were related to samples with higher reactivity. The distances between scores and the ellipse allowed the sample reactivity to be discriminated.

Notably, the samples allowed to react for 1 h were very close to the mercerized samples (blue ellipse), while those subjected to reaction times of 15 and 30 min differed considerably, allowing better discrimination of the sample reactivity. Scores representing reaction times of 15 and 30 min showed that, at 80 °C, the order of conversion was WCK > WCS, CLC. The scores for CLC were the most distant from the blue ellipse, indicating that the conversion after 15 and 30 min was low. Different cellulose samples clearly required different mercerization conditions. From this perspective, this study aids the prediction and adjustment of process parameters for mercerization. Although PCA analysis showed the similarity among the samples, it was difficult in this case to integrate all variables in a single score plot. Therefore, different conditions influencing cellulose reactivity should be studied simultaneously and correlated to each other. For this reason, design of experiments was performed.

### 3.4. Design of Experiments

PCA of the PXRD data showed that the CII% value at a fixed temperature was influenced by the specific treatment and processing time. By performing multivariate analysis, the simultaneous influence of temperature (T) and time (t) on the process could be assessed and used to optimize the mercerization conditions and enhance the process efficiency. This approach has already been successful employed to improve industrial processes, such as wastewater treatment [69], grinding optimization [70] and biological processes [71] in the production of biolubricants [72] and other areas of industrial interest [73].

**Figure 8:**
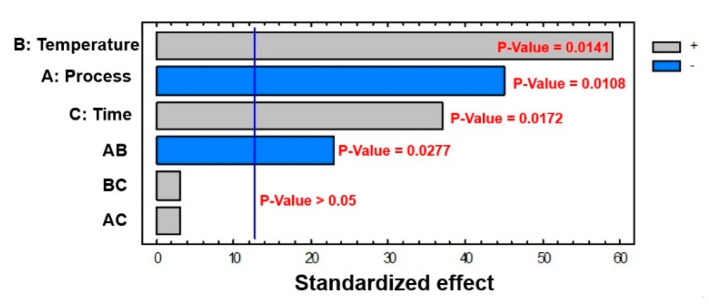
Pareto chart for conversion related to the WCK and WCS processes.

Each of the three mercerization procedures discussed above were subjected to multivariate analysis. To demonstrate the power of the multivariate approach, we have reported the analysis of mercerization involving only wood celluloses WCK and WCS. The temperature range considered was 25 °C < T < 80 °C, while the reaction time range was 15 min < t < 48 h. Multivariate analysis of the factors, as summarized in the Pareto chart in Figure 8, showed that temperature, process, time, and the combination of process and temperature (AB in Figure 8) had relevant effects (*p* < 0.05). For clarity, “process” in the DoE section refers to the type of cellulose tested (WCK and WCS).

The relevant effects of temperature and time were also obtained from the PCA data, while the important effect of the process was not evident. In this respect, the WCK and WCS processes are usually described as very similar because both derive from the same raw cellulose (wood pulp cellulose). Furthermore, the relevant combined effect of process and temperature was only noted by multivariate analysis.

**Figure 9:**
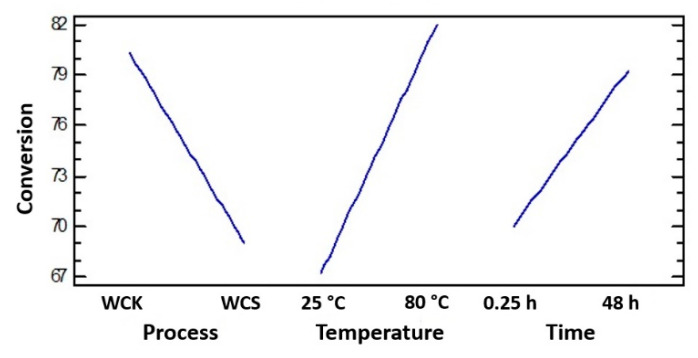
Main effect plot for conversion relative to the WCK and WCS processes.

The specific effects of each factor are better highlighted in the main effect plot shown in Figure 9.

From the main factors plot, the WCK process evidently allowed a higher cellulose conversion to be reached compared with the sulfite treatment. From analysis of the slope of each curve, the magnitude of the effects of each factor on the conversion could be compared. Temperature had the greatest impact on the conversion, followed by the process and reaction time, which showed similar slopes.

**Figure 10:**
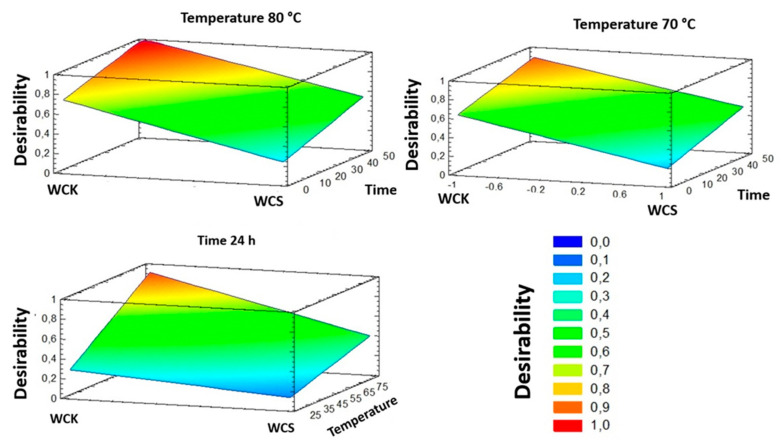
Estimated response surface for cellulose produced through the WCK and WCS processes. Temperature and time are expressed in °C and h, respectively.

To optimize these parameters and increase the efficiency of the two processes, response surface analysis was performed (Figure 10). The response surface model was implemented to maximize the CII% value. This target was expressed as a function of desirability, which ranged from 0 (corresponding to the minimum desirability and a conversion of 60%) to 1 (corresponding to a conversion of 96%).

By employing the statistical model developed and presented herein, many working conditions could be simulated and the process of choice optimized for the mercerization of wood cellulose. By setting the process temperature to 80 °C and mercerizing for at least 10 h, high conversions (96%) could only be reached for cellulose from the WCK process (Figure 10, top left) while, for WCS, the maximum desirability reached was about 60%, corresponding to 79% conversion, even after mercerization for 48 h at 80 °C. The response surface model implemented showed that the maximum desirability could be reached by the WCK process at 70 °C (Figure 9, top right). In fact, 70 °C was the minimum temperature that led to the maximum possible conversion (96%). Furthermore, the model allowed the mercerization time to be optimized. The simulation reported in Figure 10 (below left) showed that 24 h was the minimum process time needed to reach the maximum conversion.

Further simulations based on the present model are reported in the Supporting Information (Figures S4–S13 and Tables S4–S10).

## 4. Conclusions

A set of analytical tools for estimating and improving the efficiency of the mercerization process applied to industrial cellulose samples was presented. Three industrial celluloses were mercerized under different conditions, and the conversion from CI to CII (CII%) and crystallinity (CI%) were determined by PXRD. The combination of tablet sample preparation and high-quality X-ray data acquisition in transmission mode was conveniently exploited as the input for PCA and multivariate analysis of the PXRD data by DoE. This approach allowed all relevant parameters influencing the conversion in industrial mercerization processes to be determined. Furthermore, by combining the statistical results with an implemented desirability function, a suitable model for the optimization of mercerization efficiency was formulated by performing response surface analysis. In particular, a specific tool for tuning the conversion of different wood celluloses by properly modifying the temperature, reaction time, and specific process was presented and discussed. The multivariate model proposed allowed relevant hidden differences between celluloses, which apparently have similar characteristics, to be determined.

## Figures and Tables

**Table 1:** Main parameters of the studied cellulose.

Cellulose	C.I.%	DP ^a^	Hemicellulose Content%	Particle Size (m)
WCK	58	500–700	19	500
WCS	54	1000–1300	9	500
CLC	73	1000–5000	-	500

^a^ DP values were obtained from supplier.

## Supplementary Materials

The following are available online at https://www.mdpi.com/2073-4360/12/7/1559/s1, Figure S1. XRD peak deconvolution of WCS; Figure S2. XRD peak deconvolution of WCK; Figure S3. XRD diffractogramms of CI (top) and CII (bottom); Figure S4. Estimated surface response after 1 h of mercerization; Figure S5. Estimated surface response after 6 h of mercerization; Figure S6. Estimated surface response after 12 h of mercerization; Figure S7. Estimated surface response after 18 h of mercerization; Figure S8. Estimated surface response after 24 h of mercerization; Figure S9. Estimated surface response after 36 h of mercerization; Figure S10. Estimated surface response for the mercerization conducted at 25 °C; Figure S11. Estimated surface response for the mercerization conducted at 50 °C; Figure S12. Estimated surface response for the mercerization conducted at 70 °C; Figure S13. Estimated surface response for the mercerization conducted at 80 °C; Table S1. Crystallinity (C.I.%) and conversion (CII%) values obtained from XRD for CLC; Table S2. Crystallinity (C.I.%) and conversion (CII%) values obtained from XRD for WCS; Table S3. Crystallinity (C.I.%) and Conversion (CII%) values obtained from XRD for WCK; Table S4. Independent and dependent variables considered; Table S5. Response; Table S6. Randomized order and condition of the experiments; Table S7. Analysis of the factors; Table S8. ANOVA table; Table S9. Main data for the multiple response implementation; Table S10. Data relative to the implementation of the desirability function.

## References

[1] Fan L., Gharpuray M.M., Lee Y. (1987). Nature of cellulosic material. Cellulose Hydrolysis.

[2] Klemm D., Heublein B., Fink H.P., Bohn A. (2005). Cellulose: Fascinating biopolymer and sustainable raw material. Angew. Chem. Int. Ed..

[3] Nishiyama Y., Langan P., Chanzy H. (2002). Crystal Structure and Hydrogen-Bonding System in Cellulose Iβ from Synchrotron X-ray and Neutron Fiber Diffraction. J. Am. Chem. Soc..

[4] Klemm D., Philip B., Heinze T., Heinze U., Wagenknecht W. (1998). Comprehensive Cellulose Chemistry, Volume 2: Derivatization of Cellulose.

[5] Zugenmaier P. (2008). Crystalline Cellulose and Cellulose Derivatives: Characterization and Structures.

[6] Stone B. (2001). Cellulose: Structure and Distribution. Biotechnology.

[7] Atalla R.H., Vanderhart D.L. (1984). Native Cellulose: A Composite of Two Distinct Crystalline Forms. Science.

[8] Mozdyniewicz D.J., Nieminen K., Sixta H. (2013). Alkaline Steeping of Dissolving Pulp. Part I: Cellulose Degradation Kinetics. Cellulose.

[9] Albán Reyes D.C., Gorzsás A., Stridh K., de Wit P., Sundman O. (2017). Alkalization of dissolving cellulose pulp with highly concentrated caustic at low NaOH stoichiometric excess. Carbohydr. Polym..

[10] Fischer S., Thümmler K., Volkert B., Hettrich K., Schmidt I., Fischer K. (2008). Properties and Applications of Cellulose Acetate. Macromol. Symp..

[11] Tait C.W., Vetter R.J., Swanson J.M., Debye P. (1951). Physical characterization of cellulose xanthate in solution. J. Polym. Sci..

[12] Ferro M., Castiglione F., Panzeri W., Dispenza R., Santini L., Karlsson H.J., De Wit P.P., Mele A. (2017). Non-destructive and direct determination of the degree of substitution of carboxymethyl cellulose by HR-MAS ^13^C NMR spectroscopy. Carbohydr. Polym..

[13] Hollabaugh C.B., Burt L.H., Walsh A.P. (1945). Carboxymethylcellulose. Uses and Applications. Ind. Eng. Chem..

[14] Wustenberg T. (2014). Sodium Carboxymethylcellulose. Cellulose and Cellulose Derivatives in the Food Industry.

[15] Horii F., Hirai A., Kitamaru R. (1987). Cross-Polarization-Magic Angle Spinning Carbon-13 NMR Approach to the Structural Analysis of Cellulose. The Structures of Cellulose.

[16] Ye D., Farriol X. (2005). Improving accessibility and reactivity of celluloses of annual plants for the synthesis of methylcellulose. Cellulose.

[17] Maurer A., Fengel D. (1992). Parallel orientation of the molecular chains in cellulose I and cellulose II deriving from higher plants. Holz Roh Werkst..

[18] Verlhac C., Dedier J., Chanzy H. (1990). Availability of surface hydroxyl groups in valonia and bacterial cellulose. J. Polym. Sci. Part A Polym. Chem..

[19] Köpcke V. (2008). Improvement on Cellulose Accessibility and Reactivity of Different Wood Pulps. Ph.D. Thesis.

[20] Kolpak F., Francis J. (1976). Determination of the Structure of Cellulose II. Macromolecules.

[21] Kolpak F.J., Weih M., Blackwell J. (1978). Mercerization Cellulose: 1. Determination of the structure of mercerized cotton. Polymer.

[22] Dinand E., Vignon M., Chanzy H., Heux L. (2002). Mercerization of primary wall cellulose and its implication for the conversion of cellulose I to cellulose II. Cellulose.

[23] Gupta P.K., Uniyal V., Naithani S. (2013). Polymorphic transformation of cellulose i to cellulose II by alkali pretreatment and urea as an additive. Carbohydr. Polym..

[24] Nishimura H., Okano T., Sarko A. (1991). Mercerization of cellulose. Crystal and molecular structure of Na-cellulose I. Macromolecules.

[25] Langan P., Nishiyama Y., Chanzy H. (2001). X-ray Structure of Mercerized Cellulose II at 1 Å Resolution. Biomacromolecules.

[26] Raymond S., Kvick A., Chanzy H. (1995). The structure of cellulose II: A revisit. Macromolecules.

[27] Langan P., Nishiyama Y., Chanzy H. (1999). A Revised Structure and Hydrogen-Bonding System in Cellulose II from a Neutron Fiber Diffraction Analysis. J. Am. Chem. Soc..

[28] Jeffries R. (1964). The amorphous fraction of cellulose and its relation to moisture sorption. J. Appl. Polym. Sci..

[29] Pönni R., Rautkari L., Hill C.A.S., Vuorinen T. (2014). Accessibility of hydroxyl groups in birch kraft pulps quantified by deuterium exchange in D_2_O vapor. Cellulose.

[30] Jin E., Guo J., Yang F., Zhu Y., Song J., Jin Y., Rojas O.J. (2016). On the polymorphic and morphological changes of cellulose nanocrystals (CNC-I) upon mercerization and conversion to CNC-II. Carbohydr. Polym..

[31] Halonen H., Larsson P.T., Iversen T. (2013). Mercerized cellulose biocomposites: A study of influence of mercerization on cellulose supramolecular structure, water retention value and tensile properties. Cellulose.

[32] Wang H., Farooq A., Memon H. (2020). Influence of cotton fiber properties on the microstructural characteristics of mercerized fibers by regression analysis. Wood Fiber Sci..

[33] Younesi M., Wu X., Akkus O. (2019). Controlled mercerization of bacterial cellulose provides tunability of modulus and ductility over two orders of magnitude. J. Mech. Behav. Biomed. Mater..

[34] Gralén N. (1955). Cellulose and cellulose derivatives. J. Polym. Sci..

[35] Ranby B. (1952). The Mercerisation of Cellulose. I. A Thermodynamic Discussion. Acta Chem. Scand..

[36] Shen L., Worrell E., Patel M.K. (2010). Environmental impact assessment of man-made cellulose fibres. Resour. Conserv. Recycl..

[37] George M., Bressler D.C. (2017). Comparative evaluation of the environmental impact of chemical methods used to enhance natural fibres for composite applications and glass fibre based composites. J. Clean. Prod..

[38] Keshk S.M.A.S. (2015). Effect of different alkaline solutions on crystalline structure of cellulose at different temperatures. Carbohydr. Polym..

[39] Nomura S., Kugo Y., Erata T. (2020). 13C NMR and XRD studies on the enhancement of cellulose II crystallinity with low concentration NaOH post-treatments. Cellulose.

[40] Sisson W., Saner W. (2002). The Effect of the Temperature and the Concentration of Sodium Hydroxide on the X-ray Diffraction Behavior of Raw and of Degraded Cotton. J. Phys. Chem..

[41] Liu Y., Hu H. (2008). X-ray diffraction study of bamboo fibers treated with NaOH. Fibers Polym..

[42] Colom X., Carrillo F. (2002). Crystallinity changes in lyocell and viscose-type fibres by caustic treatment. Eur. Polym. J..

[43] Reyes D., Skoglund N., Svedberg A., Eliasson B., Sundman O. (2016). The influence of different parameters on the mercerisation of cellulose for viscose production. Cellulose.

[44] Ottani S., Riello P., Polizzi S. (1993). Complete sets of factors for absorption correction and air scattering subtraction in X-ray powder diffraction of loosely packed sample. Powder Diffr..

[45] Sluiter A., Hames B., Ruiz R., Scarlata C., Sluiter J., Templeton D., Crocker D.L.A.P. (2008). Determination of Structural Carbohydrates and Lignin in Biomass. Natl. Renew. Energy Lab..

[46] Park S., Johnson D.K., Ishizawa C.I., Parilla P.A., Davis M.F. (2009). Measuring the crystallinity index of cellulose by solid state 13C nuclear magnetic resonance. Cellulose.

[47] Ju X., Bowden M., Brown E.E., Zhang X. (2015). An improved X-ray diffraction method for cellulose crystallinity measurement. Carbohydr. Polym..

[48] Zhao H., Kwak J.H., Conrad Zhang Z., Brown H.M., Arey B.W., Holladay J.E. (2007). Studying cellulose fiber structure by SEM, XRD, NMR and acid hydrolysis. Carbohydr. Polym..

[49] Leppänen K., Andersson S., Torkkeli M., Knaapila M., Kotelnikova N., Serimaa R. (2009). Structure of cellulose and microcrystalline cellulose from various wood species, cotton and flax studied by X-ray scattering. Cellulose.

[50] Andersson S., Serimaa R., Paakkari T., SaranpÄÄ P., Pesonen E. (2003). Crystallinity of wood and the size of cellulose crystallites in Norway spruce (Picea abies). J. Wood Sci..

[51] Terinte N., Ibbett R., Schuster K.C. (2011). Overview on Native Cellulose and Microcrystalline Cellulose I Structure Studied By X-Ray Diffraction (Waxd): Comparison Between Measurement Techniques. Lenzing. Ber..

[52] French A.D. (2014). Idealized powder diffraction patterns for cellulose polymorphs. Cellulose.

[53] Wojdyr M. (2010). Fityk: A general-purpose peak fitting program. J. Appl. Crystallogr..

[54] Chen R., Jakes K.A., Foreman D.W. (2004). Peak-fitting analysis of cotton fiber powder X-ray diffraction spectra. J. Appl. Polym. Sci..

[55] Thygesen A., Oddershede J., Lilholt H., Thomsen A.B., Ståhl K. (2005). On the determination of crystallinity and cellulose content in plant fibres. Cellulose.

[56] Oh S.Y., Dong I.Y., Shin Y., Hwan C.K., Hak Y.K., Yong S.C., Won H.P., Ji H.Y. (2005). Crystalline structure analysis of cellulose treated with sodium hydroxide and carbon dioxide by means of X-ray diffraction and FTIR spectroscopy. Carbohydr. Res..

[57] Xia J., Wishart D. (2016). Using MetaboAnalyst 3.0 for Comprehensive Metabolomics Data Analysis. Curr. Protoc. Bioinform..

[58] Box G., Stuart Hunter J., Hunter W.G. (2005). Statistics for Experimenters: Design, Innovation, and Discovery.

[59] Borysiak S., Garbarczyk J. (2003). Applying the WAXS method to estimate the supermolecular structure of cellulose fibres after mercerisation. Fibres Text. East. Eur..

[60] Mansikkamäki P., Lahtinen M., Rissanen K. (2007). The conversion from cellulose I to cellulose II in NaOH mercerization performed in alcohol–water systems: An X-ray powder diffraction study. Carbohydr. Polym..

[61] Mansikkamäki P., Lahtinen M., Rissanen K. (2005). Structural changes of cellulose crystallites induced by mercerisation in different solvent systems; determined by powder X-ray diffraction method. Cellulose.

[62] Duchemin B.J.C. (2015). Mercerisation of cellulose in aqueous NaOH at low concentrations. Green Chem..

[63] Revol J.F., Dietrich A., Goring D.A.I. (1987). Effect of mercerization on the crystallite size and crystallinity index in cellulose from different sources. Can. J. Chem..

[64] Le Moigne N., Bikard J., Navard P. (2010). Rotation and contraction of native and regenerated cellulose fibers upon swelling and dissolution: The role of morphological and stress unbalances. Cellulose.

[65] Tanimoto T., Nakano T. (2013). Side-chain motion of components in wood samples partially non-crystallized using NaOH-water solution. Mater. Sci. Eng. C.

[66] Fink H.-P., Hofmann D., Purz H.J. (1990). Zur Fibrillarstruktur nativer Cellulose. Acta Polym..

[67] Abdi H., Williams L.J. (2010). Principal component analysis. Wiley Interdiscip. Rev. Comput. Stat..

[68] Jolliffe I.T. (2002). Principal Component Analysis.

[69] Dopar M., Kusic H., Koprivanac N. (2011). Treatment of simulated industrial wastewater by photo-Fenton process. Part I: The optimization of process parameters using design of experiments (DOE). Chem. Eng. J..

[70] Alagumurthi N., Palaniradja K., Soundararajan V. (2006). Optimization of Grinding Process Through Design of Experiment (DOE)—A Comparative Study. Mater. Manuf. Process..

[71] Mandenius C.-F., Brundin A. (2008). Bioprocess optimization using design-of-experiments methodology. Biotechnol. Prog..

[72] Vlahopoulou G., Petretto G.L., Garroni S., Piga C., Mannu A. (2018). Variation of density and flash point in acid degummed waste cooking oil. J. Food Process. Preserv..

[73] Weissman S.A., Anderson N.G. (2015). Design of Experiments (DoE) and Process Optimization. A Review of Recent Publications. Org. Process Res. Dev..

