# Supplementary material for: An Integrated Approach to Optimizing Cellulose Mercerization
Source: Polymers (Basel). 2020 Jul 14;12(7):1559. doi: 10.3390/polym12071559 (PMC7407994; doi:10.3390/polym12071559)
Supplement: Supplementary file 1 [file polymers-12-01559-s001.pdf]

An integrated approach to optimizing cellulose mercerization

Monica Ferro,[*a] Alberto Mannu,[a] Walter Panzeri,[b] C. H. J. Theeuwen[c] and Andrea Mele*[a,b]

a. Department of Chemistry, Materials and Chemical Engineering "G. Natta", Politecnico di Milano, Piazza L. da Vinci 32, 20133 Milano, Italy E-mail: monica.ferro@polimi.it, andrea.mele@polimi.it.

[b]*CNR – SCITEC, Istituto di Scienze e Tecnologie Chimiche, Via Alfonso Corti 12, 20133 Milano, Italy*

[c]*Nouryon Chemicals bv, Westervoortsedijk 73, 6827 AV Arnhem, The Netherlands*

monica.ferro@polimi.it , andrea.mele@polimi.it.

SUPPLEMENTARY INFORMATION - Examples of peak fitting

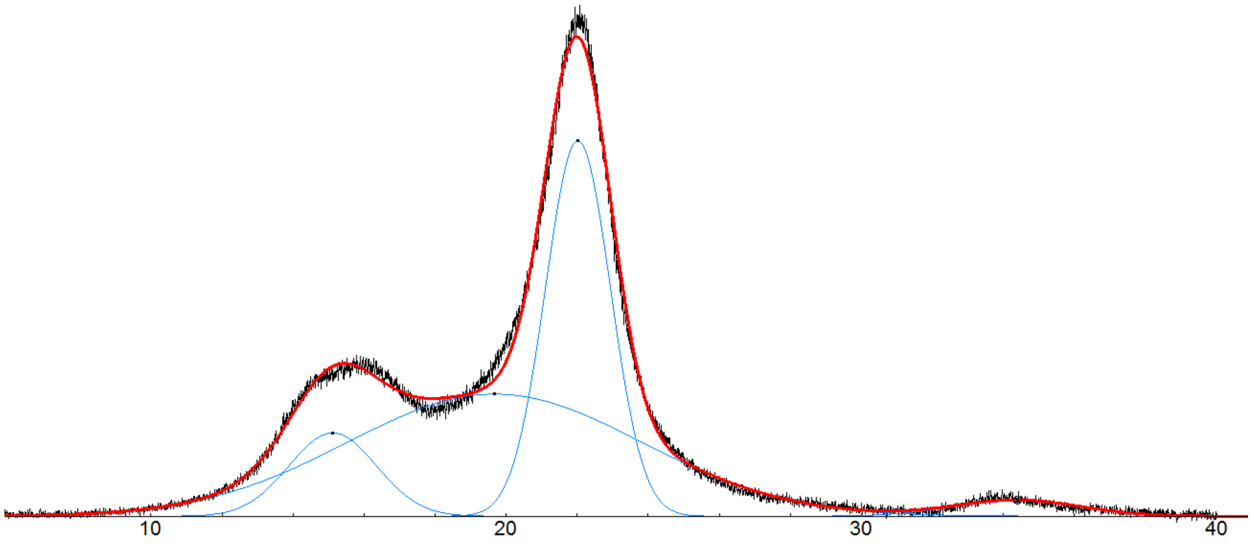

Example 1. XRD peak deconvolution of WCK using 4 gaussian functions. Black: experimental pattern, Blue: Gaussian curved used, Red: simulated spectrum.

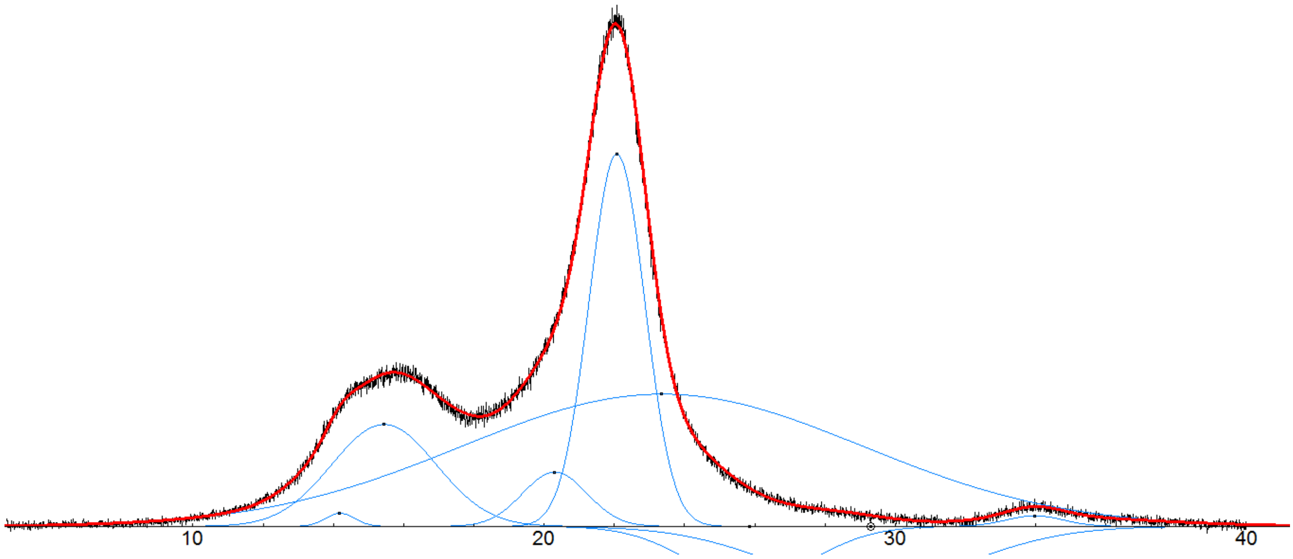

Example 2. XRD peak deconvolution of WCK using 9 gaussian functions. Black: experimental pattern, Blue: Gaussian curved used, Red: simulated spectrum.

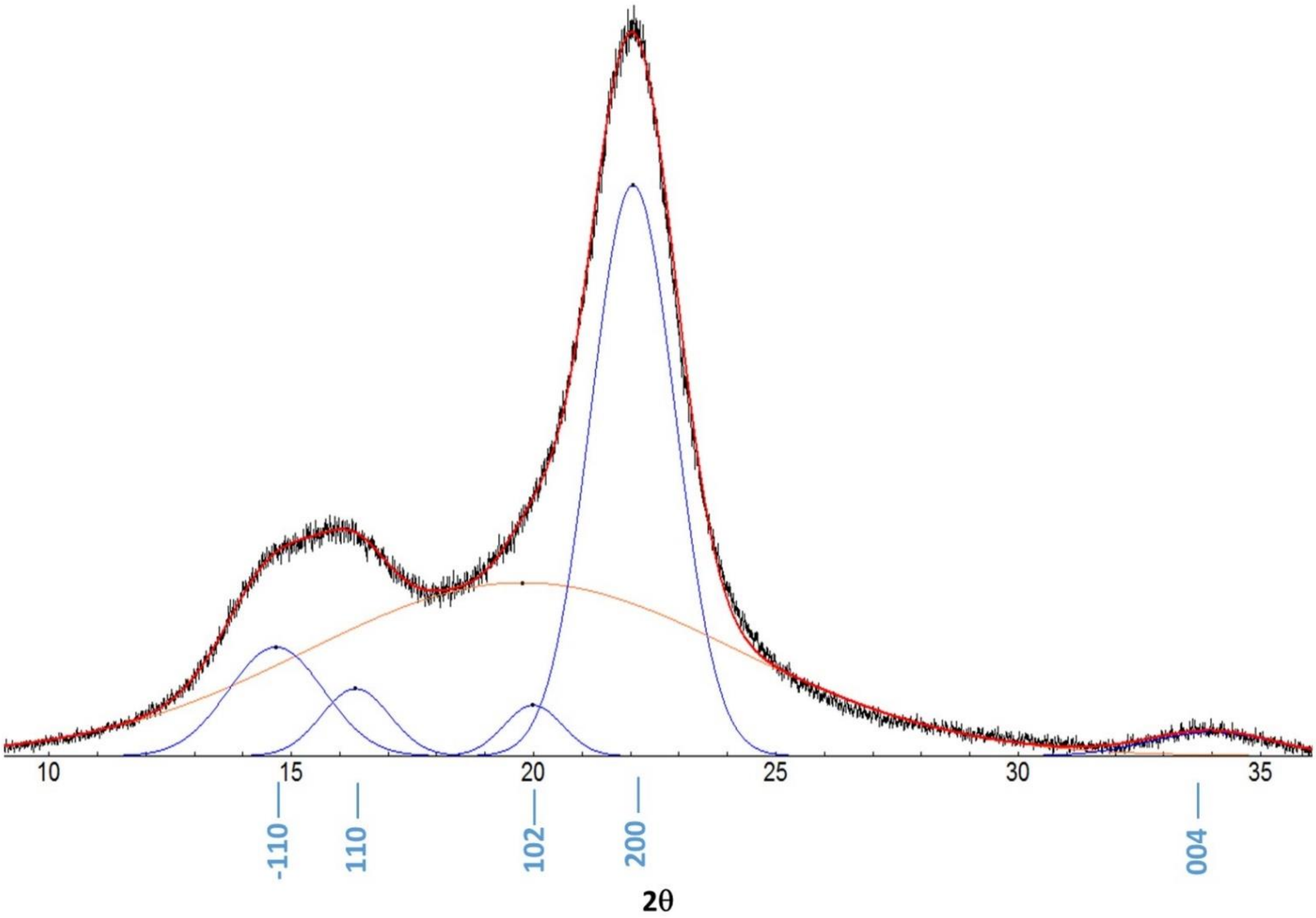

Figure S1. XRD peak deconvolution of WCS

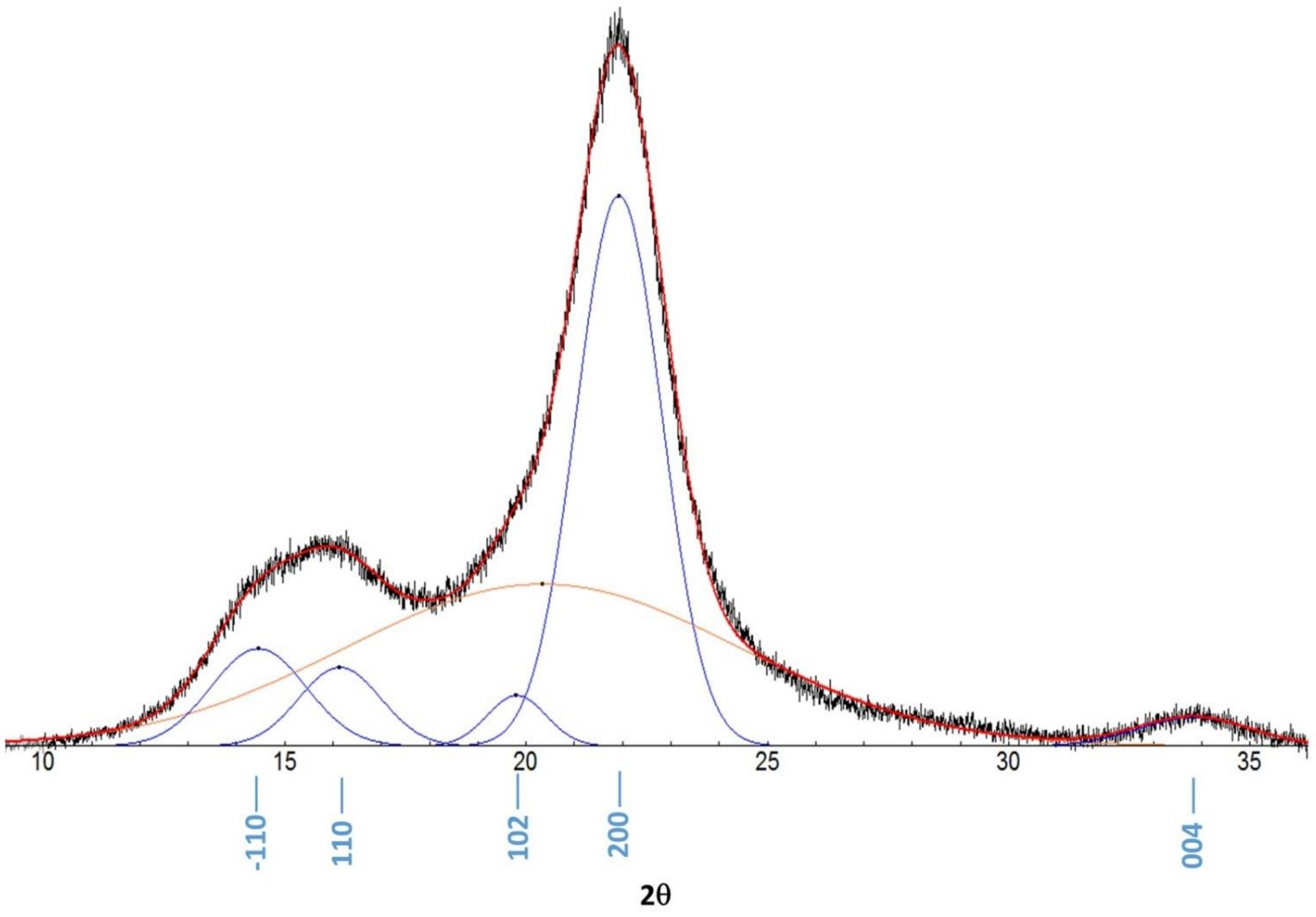

Figure S2. XRD peak deconvolution of WCK

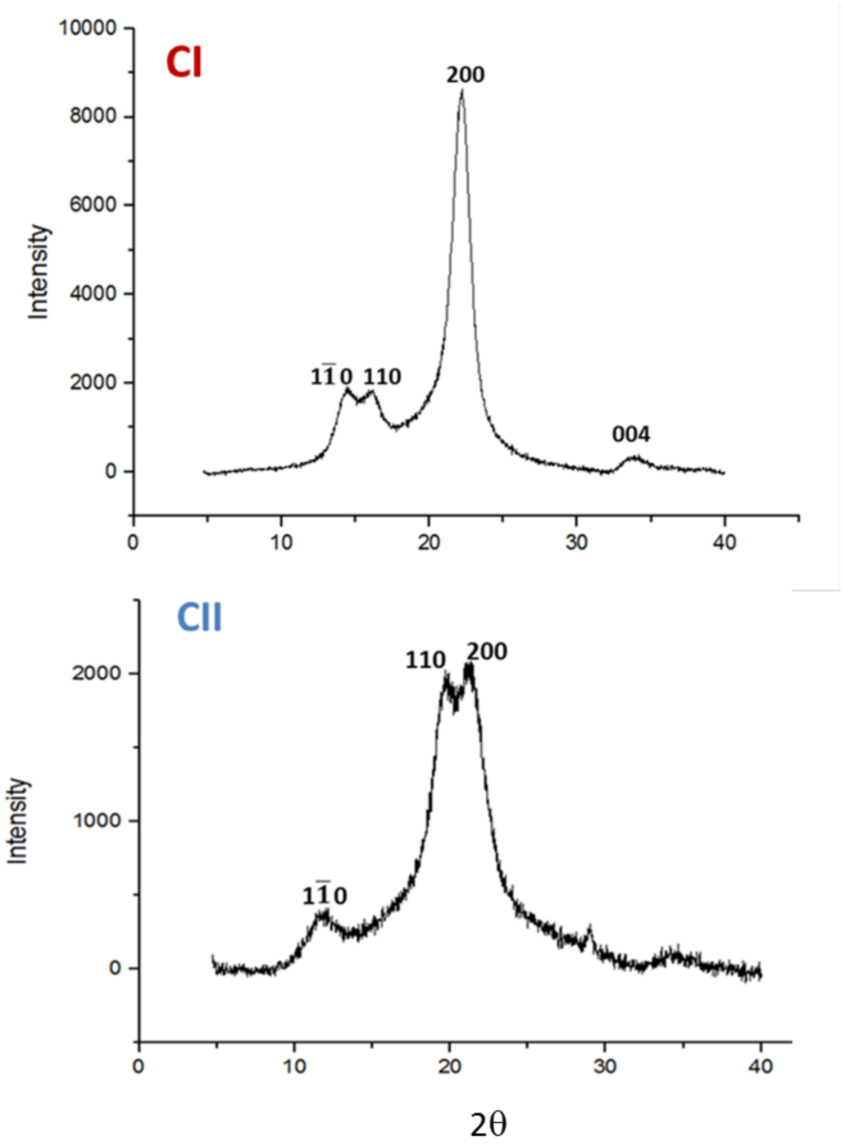

Figure S3. XRD diffractogramms of CI (top) and CII (bottom).

Table S1. Crystallinity (C.I.%) and Conversion (CII%) values obtained from XRD for CLC

Temp (°C)		*0*	*15'*	*30'*	*1h*	*48h*
25 °C	C.I.%	73	60	60	57	57
	CII%	0	49	54	54	58
40 °C	C.I.%	73	58	58	60	60
	CII%	0	50	50	50	60
60 °C	C.I.%	73	49	49	46	46
	CII%	0	58	62	63	72
80°C	C.I.%	73	49	47	46	46
	CII%	0	64	65	70	80

Table S2. Crystallinity (C.I.%) and Conversion (CII%) values obtained from XRD for WCS

Temp (°C)		*0*	*15'*	*30'*	*1h*	*48h*
25 °C	C.I.%	54	51	50	49	49
	CII%	0	60	60	61	69
40 °C	C.I.%	54	49	45	44	43
	CII%	0	63	64	71	71
60 °C	C.I.%	54	43	43	43	43
	CII%	0	62	69	75	81
80°C	C.I.%	54	45	44	43	43
	CII%	0	68	70	75	79

Table S3. Crystallinity (C.I.%) and Conversion (CII%) values obtained from XRD for WCK

Temp (°C)		0	15’	30’	1h	48h
25 °C	C.I.%	58	51	51	46	46
	CII%	0	66	67	71	74
40 °C	C.I.%	58	51	51	48	46
	CII%	0	70	75	82	88
60 °C	C.I.%	58	50	47	46	39
	CII%	0	87	88	89	96
80°C	C.I.%	58	44	44	41	39
	CII%	0	86	88	89	95

Comparison between WCK and WCS

Full Factorial Experimental Design

A two levels full factorial experimental design n^k was implemented for analyse the behaviour of three independent variables (factors k) and one dependent variable (response), as shown in Tables S4 and S5.

Table S4: independent and dependent variables considered.

Factors k	**Levels**		***Unit***
	Low (-1)	***High (+1)***	
**Process**	WCK	WCS	
**Temperature**	25	80	°C
**Time**	0.25	48	h

The response conversion was expressed as percentage of cellulose of type II formed, CII%.

Table S5: Response

**Response**	**Unit**
Conversion	CII%

The factorial combination of the three independent variables generated 9 experiments (2^3), which have been conducted following the full randomized order reported in Table S6.

Table S6: Randomized order and condition of the experiments.

Experiment	Process	Temperature	Time	CII%
1	WCK	80	0.25	86
2	WCS	80	48	79
3	WCK	25	0.25	66
4	WCS	80	48	95
5	WCK	80	0.25	68
6	WCS	25	48	74
7	WCK	25	0.25	60
8	WCK	25	48	69

Analysis of the effects and interactions

In table S7 each of the estimated effects and interactions are reported with the corresponding standard error.

Table S7: analysis of the factors

Effect	Estimate	Stnd. Error	V.I.F.
average	74,625	0,125	
A:Process	-11,25	0,25	1,0
B:Temperature	14,75	0,25	1,0
C:Time	9,25	0,25	1,0
AB	-5,75	0,25	1,0
AC	0,75	0,25	1,0
BC	0,75	0,25	1,0

The standard error for each of the factors and combination is reported in Table S7 (column Stnd. Error). The term V.I.F. indicates the largest variance inflation factor. V.I.F = 1 for all of the factors indicates a perfectly orthogonal design for all the factors. These data are usually employed for building the Pareto chart (reported and discussed in the main manuscript, figure S5).

The analysis of the variance is reported in Table S8.

Table S8: ANOVA table

Source	*Sum of Squares*	*Df*	*Mean Square*	*F-Ratio*	*P-Value*
A:Process	253,125	1	253,125	2025,00	0,0141
B:Temperature	435,125	1	435,125	3481,00	0,0108
C:Time	171,125	1	171,125	1369,00	0,0172
AB	66,125	1	66,125	529,00	0,0277
AC	1,125	1	1,125	9,00	0,2048
BC	1,125	1	1,125	9,00	0,2048
Total error	0,125	1	0,125		
Total (corr.)	927,875	7			

R-squared = 99,9865 percent
R-squared (adjusted for d.f.) = 99,9057 percent
Standard Error of Est. = 0,353553
Mean absolute error = 0,125

In the ANOVA table the overall contribute to all the factors and their combinations to the variance is partitioned, each part indicating the contribution to the variance of the individual factors or their combination. For each contribution, its statistical significance was assessed by comparing the mean square against an estimate of the experimental error. In our case, four effects lead to p-values less than 0,05, indicating that they are significantly different from zero at the 95,0% confidence level.

The R-Squared statistic indicates that the model as fitted explains 99,9865% of the variability in CII%. The adjusted R-squared statistic, which is more suitable for comparing models with different numbers of independent variables, is 99,9057%. The standard error of the estimate shows the

standard deviation of the residuals to be 0,353553. The mean absolute error (MAE) of 0,125 is the average value of the residuals.

Multiple Response Optimization

Data variables: CII%.

Table S9: main data for the multiple response implementation.

	Desirability	*Desirability*		*Weights*	*Weights*	
Response	*Low*	*High*	*Goal*	*First*	*Second*	*Impact*
Conv	60,0	95,0	Maximize	1,0		3,0

Table S10: data relative to the implementation of the desirability function.

		Predicted	*Observed*
Row	*Conv*	*Desirability*	*Desirability*
1	86,0	0,739286	0,742857
2	79,0	0,539286	0,542857
3	66,0	0,175	0,171429
4	95,0	1,0	1,0
5	68,0	0,232143	0,228571
6	74,0	0,396429	0,4
7	60,0	0,0	0,0
8	69,0	0,260714	0,257143

The model build allows to determine the combination of experimental factors which simultaneously optimize the CII%. The lower and upper limits of this model are related to the range of CII% experimental observed. In order to implement such values into the model a desirability function was developed. It ranges from 0, which correspond to a 60 % of CII%, to 1, which indicates 96% of CII%. Thus, the model was adjusted for achieve the target “maximize CII%” and a series of surface responding plots were drawn.

The surface responding plots can be used to find the best combination of factors which achieves the overall optimum desirability. The simultaneously effect of time, process and temperature can be studied and specific targets (i.e. reduce the process time without affect the CII%, or find the best compromise between reduced temperature and time) can be achieved (Figures SI4 to SI11).

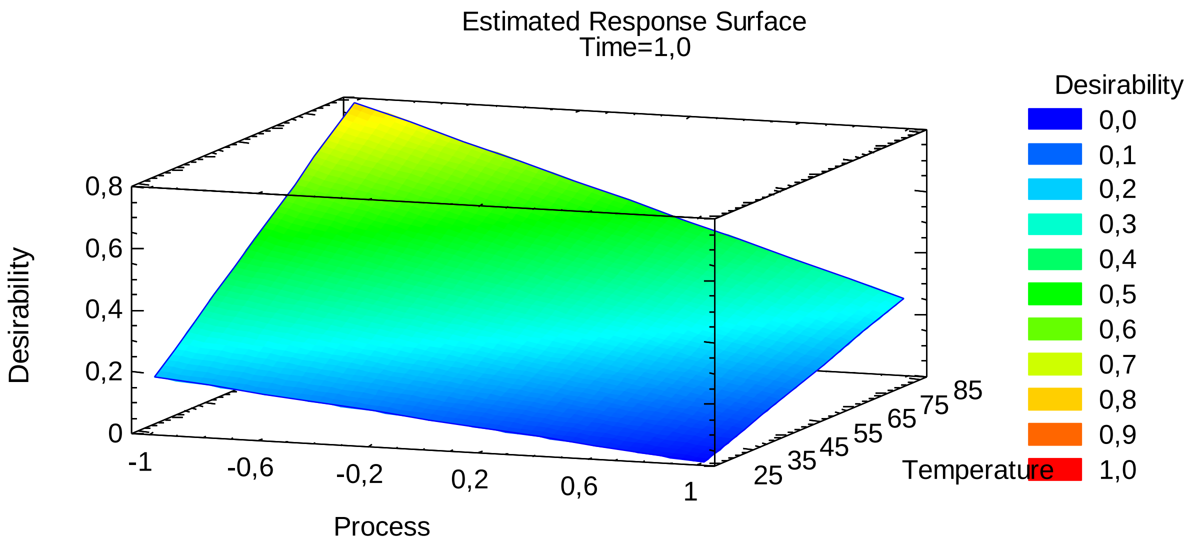

Figure S4: Estimated surface response after 1 h of mercerization.

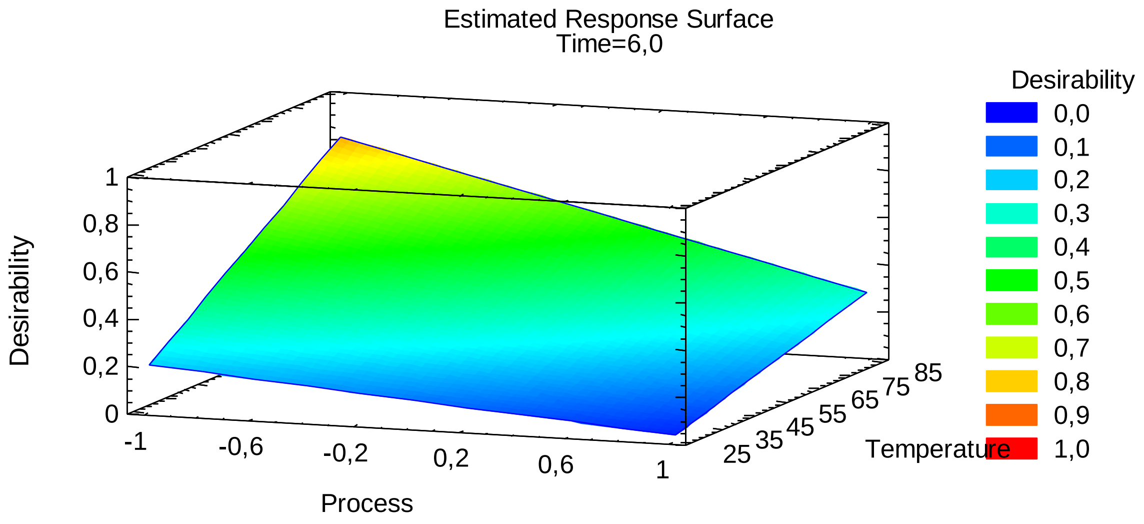

Figure S5: Estimated surface response after 6 h of mercerization.

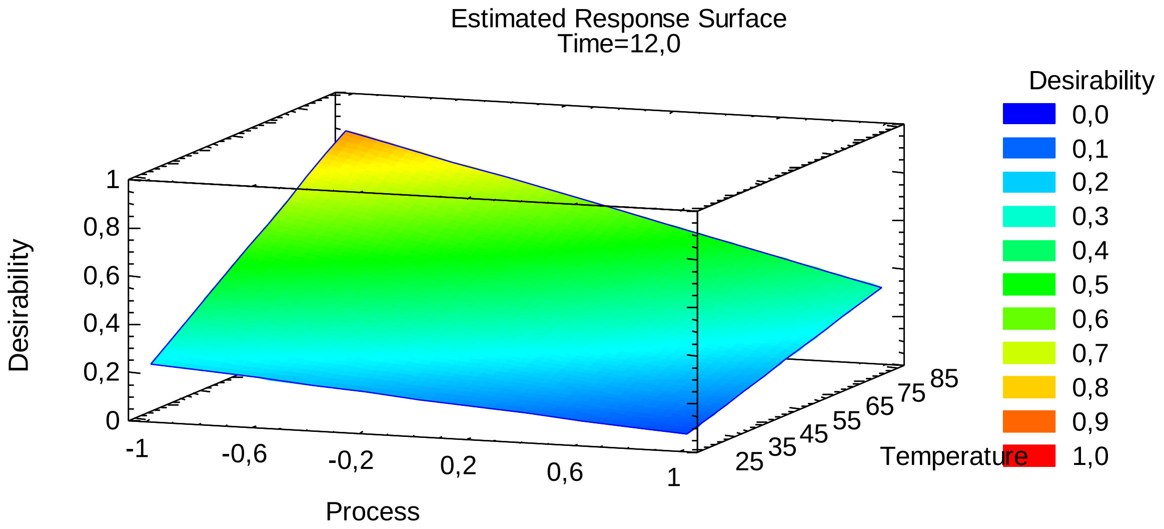

Figure S6: Estimated surface response after 12 h of mercerization.

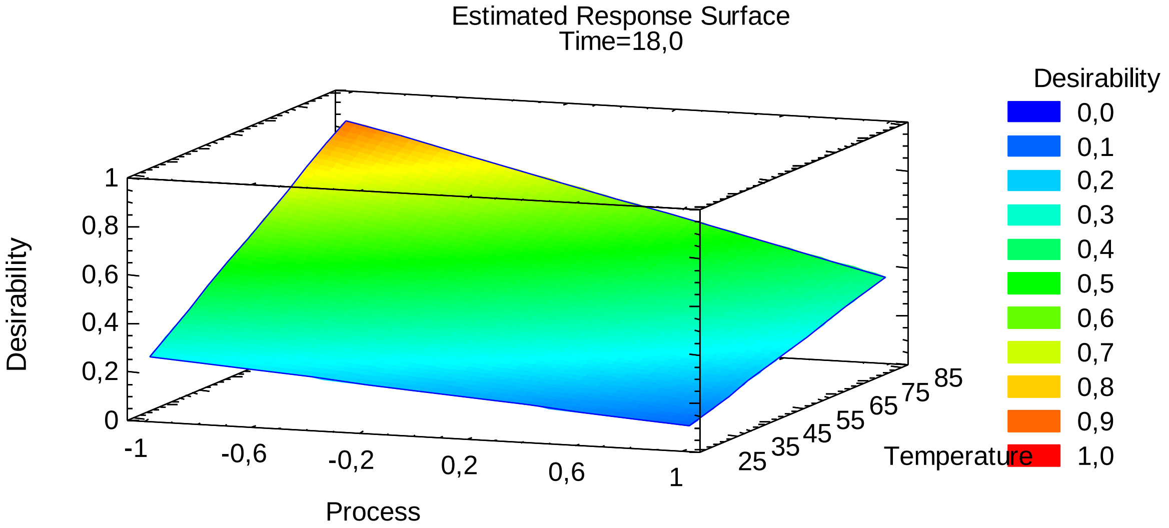

Figure S7: Estimated surface response after 18 h of mercerization.

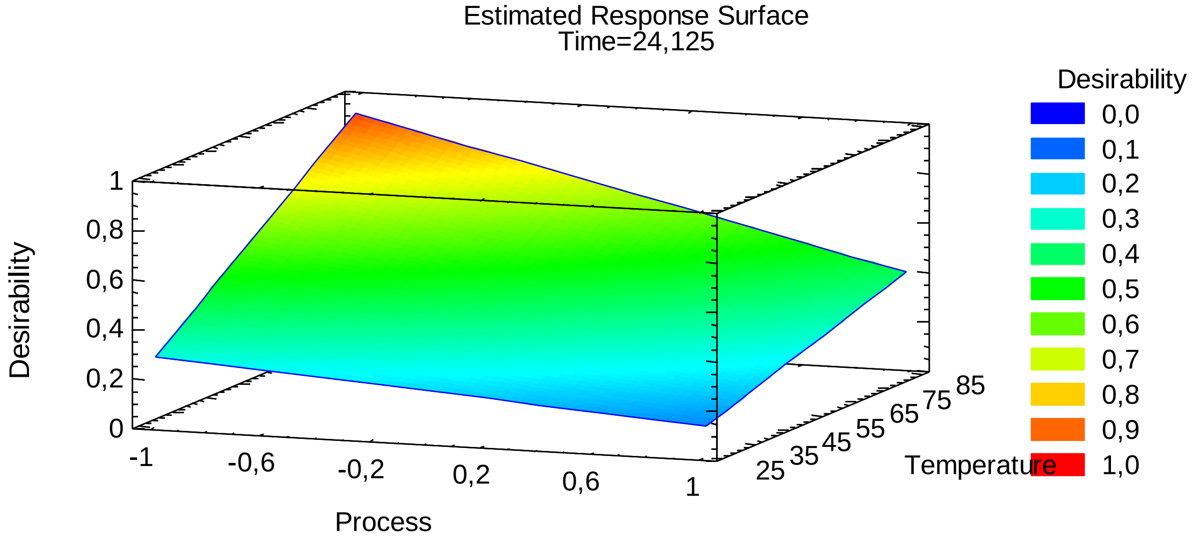

Figure S8: Estimated surface response after 24 h of mercerization.

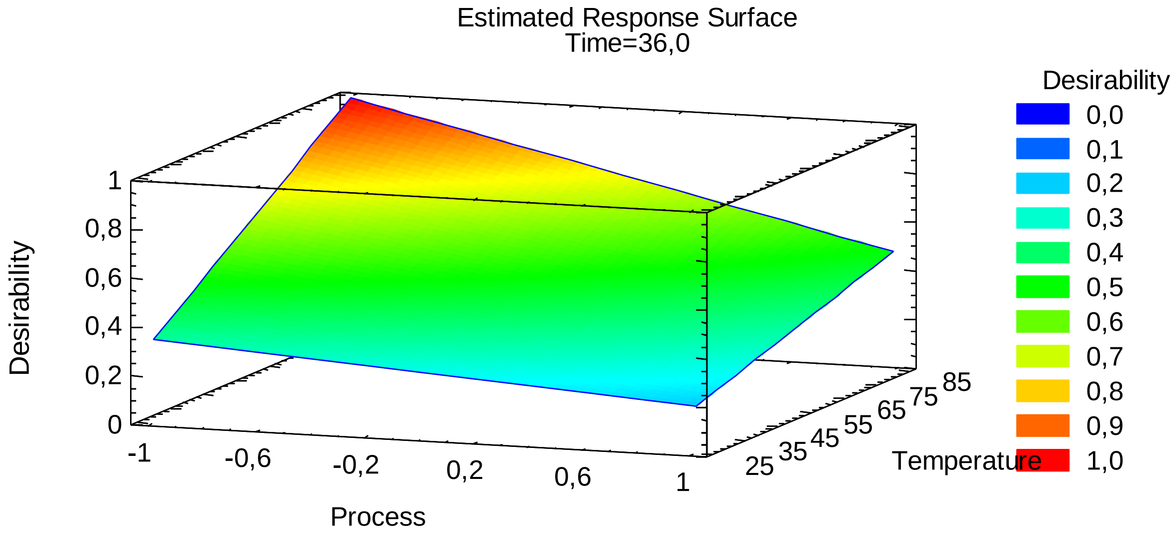

Figure S9: Estimated surface response after 36 h of mercerization.

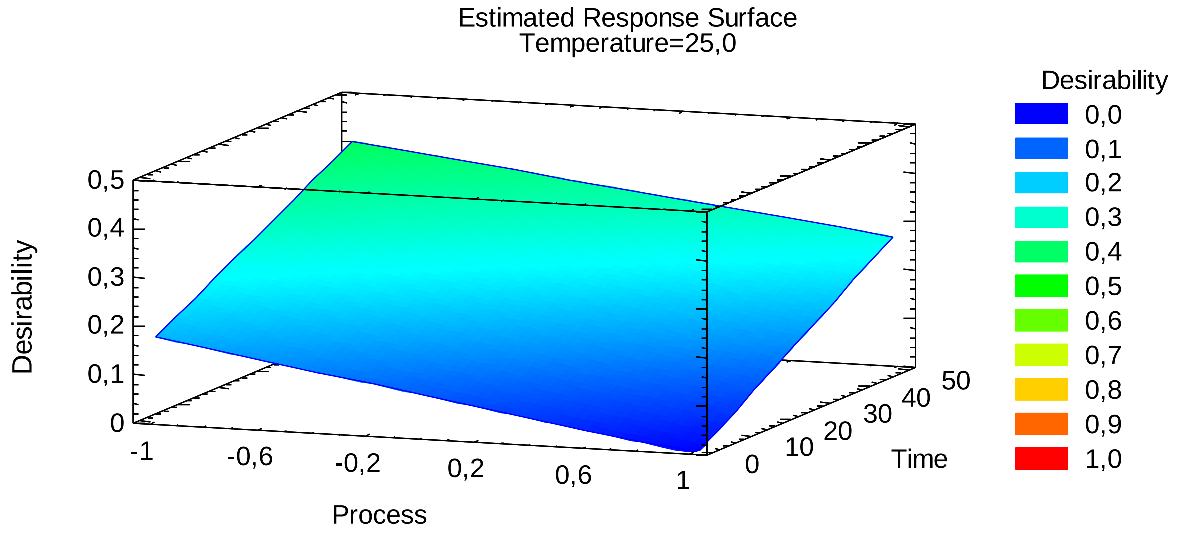

Figure S10: Estimated surface response for the mercerization conducted at 25 °C.

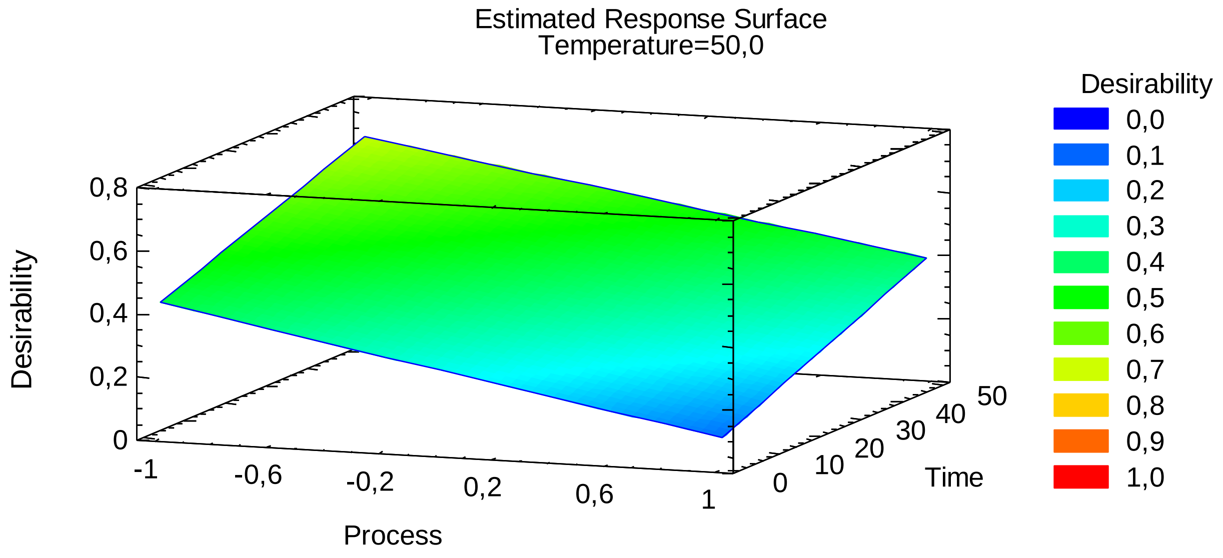

Figure S11: Estimated surface response for the mercerization conducted at 50 °C

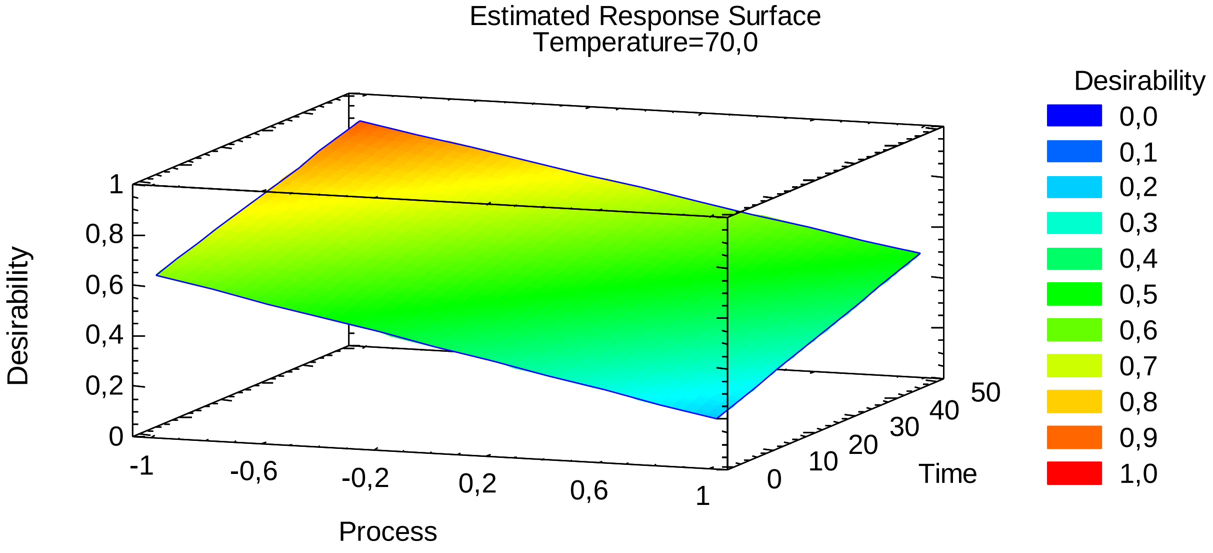

Figure S12: Estimated surface response for the mercerization conducted at 70 °C

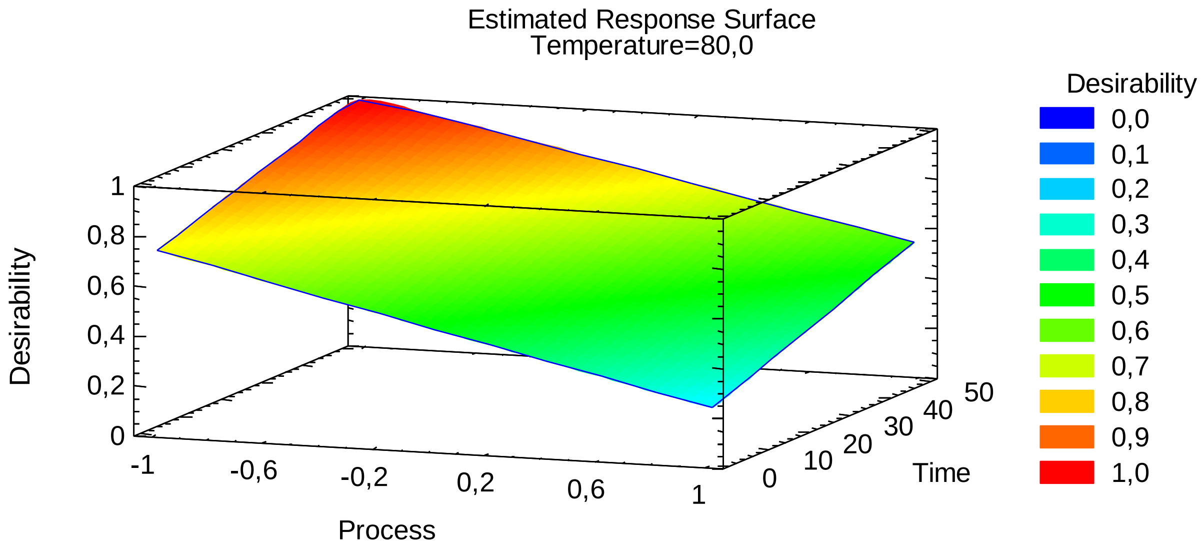

Figure S13: Estimated surface response for the mercerization conducted at 80 °C
